# Editorial: Mechanisms of Developmental and Reproductive Toxicology of Ultrafine and Nano-Sized Particles

**DOI:** 10.3389/ftox.2022.853506

**Published:** 2022-02-17

**Authors:** Masakazu Umezawa, Atsuto Onoda, Yasser Said El-Sayed

**Affiliations:** ^1^ Department of Materials Science and Technology, Faculty of Advanced Engineering, Tokyo University of Science, Tokyo, Japan; ^2^ Division of Toxicology and Health Science, Faculty of Pharmaceutical Sciences, Sanyo-Onoda City University, Yamaguchi, Japan; ^3^ Department of Veterinary Forensic Medicine and Toxicology, Faculty of Veterinary Medicine, Damanhour University, Damanhour, Egypt

**Keywords:** nanomaterials, fine particle matter (PM2.5), maternal exposure, infertility, neurotoxicity, immunotoxicity, epigenetics, antioxidant

Fine particulate matter, i.e., particles with a diameter smaller than 2.5 μm (PM2.5), is still a global issue in public health (Li et al., 2015; Lelieveld et al., 2015). Particle toxicity has been studied for coal mining dust since the 19th Century, combustion-derived particles since the Industrial Revolution, and diesel exhaust particles constituting the main component of PM2.5 since the 20th Century (Riediker et al., 2019). One health effect that has been focused in emerging evidence is that the developing fetus is susceptible to atmospheric PM2.5 containing nanoparticles. In humans, exposure to ambient particulate air pollution during pregnancy has been associated with fetal growth perturbations, childhood asthma (Wright et al., 2021), and neurobehavior (Raz et al., 2015). Previously, the developmental effects of particulate air pollution, including diesel exhaust particles, were primarily attributed to the endocrine-disrupting effects of organic chemicals, such as polycyclic aromatic hydrocarbons, adsorbed to the particles. Within the past decade, the proposed mechanisms instead involve the particles’ properties.

In addition to ambient PM2.5 containing a large proportion of ultrafine (nanosized) particles, the emerging industry has begun to apply engineering of well-designed nanoparticles in a wide range of products, including consumer and biomedical use. The unique intrapulmonary distribution after inhalation and the translocation of nanoparticles across the placenta have been described. Developmental toxicity, following exposure of pregnant animals to nanoparticles (diesel soot, carbon black, etc.,), has been observed relative to the offspring’s male reproductive system (Yoshida et al., 2010; Kyjovska et al., 2013), the immune system (El-Sayed et al., 2015) that can link to asthma susceptibility (Hazlehurst et al., 2021), the cardiovascular system (Ni et al., 2021), and the central nervous system of the brain (Umezawa et al., 2018). Oxidative stress likely plays an important role in nanoparticle toxicity, as pre-treatment with antioxidants partially suppresses the developmental toxicity of nanoparticles (Onoda et al., 2017). Our understanding of the developmental nanoparticle toxicity mechanisms should be broadened and reviewed in detail for better risk assessment and management for protecting the health of expecting mothers and their children.

This research topic collected two review articles and two original research articles. The collected articles cover the potential mechanism of the toxicity of nanoparticles on the development of the male reproductive system, immune system, and central nervous system. The roles of endogenous antioxidative activity and epigenetics in developmental particle toxicity are also reported.

Yokota et al. reviewed the effect of maternal exposure to nanoparticles on the reproductive health of male offspring. As well, they examined the negative effects of nanoparticles on male germ cells, which require multiple epigenetic reprogramming events during their lifespan to acquire reproductive capacity by altering epigenetic regulation, which may, in turn, affect embryo development. Especially, this review provides a better understanding of the possibility of nanoparticle-induced developmental male infertility based on the spermatogenesis process and changes in small non-coding RNA expression as an early biomarker of developmental toxicity in testes by prenatal nanoparticle exposure.

Li et al. reviewed the potential contribution of a major endogenous antioxidative pathway, NRF2 pathway, in preventing developmental and reproductive toxicity of fine particles. While the potential chemoprevention of the developmental toxicity of nanoparticles has been reported via the antioxidative strategy (Onoda et al., 2015), the research on the effect of endogenous antioxidative factors on developmental nanoparticle toxicity is limited. This review shows the importance of understanding the endogenous pathway for a better preventive strategy for risk management of nanoparticles based on their potential of developmental toxicity.

Onoda et al. evaluated the impact of maternal exposure to carbon-black nanoparticles via nasal instillation in mice during 3 developmental periods (embryogenesis, organogenesis, fetal) on the spleen and thymus phenotype after birth. The main findings from this study are that changes to the immune profile of offspring are impacted most during a limited window of gestational exposure to nanoparticles during the organogenesis period (gestational days 8 and 9 in mice) and that these changes manifest in the increase of non-T/non-B immune cells in the spleen. The results suggest that the organogenesis period was the most susceptible period to CB-NP exposure concerning lymphoid tissue development. The information provided by this article will be related to future strategies for preventing developmental immunotoxicity and allergic diseases caused by particulate air pollution.

Tachibana et al. reported that TiO_2_ nanoparticle exposure during gestation results in altered DNA methylation and gene expression in the brain of mice at postnatal day 1. Bioinformatics functional analysis of differentially expressed genes highlighted systems including stem cells and morphogenesis being altered due to the exposure. The method presented in this article for extracting biologically functional information from comprehensive numerical data of epigenome and transcriptome is expected to contribute to further investigations of bio/toxicological effects of environmental factors not limited to nanoparticles.

The mechanism of developmental nanoparticle toxicity is likely to be still complex and important. We hope this article collection will make people understand comprehensively and sometimes simply the toxicological mechanisms for a more effective approach to prevent negative impacts of unintentional exposure of nanoparticles on future children’s healthy life.
